# The magnitude and predictors of cervical squamous intraepithelial lesions among women in Enugu, Nigeria: a cross-sectional study of women in a low-resource setting

**DOI:** 10.11604/pamj.2022.41.130.28173

**Published:** 2022-02-16

**Authors:** Chidimma Akudo Omeke, Joseph Tochukwu Enebe, Ananyochukwu Innocent Ugwu, Nnaemeka Thaddeus Onyishi, Moses Chukwuebuka Omeke, Nympha Onyinye Enebe, Emmanuel Obiora Izuka, Elias Chike Aniwada

**Affiliations:** 1 Department of Obstetrics and Gynaecology, Enugu State University of Science and Technology Teaching Hospital, Parklane, Enugu, Nigeria,; 2 Department of Obstetrics and Gynaecology, Enugu State University of Science and Technology, College of Medicine/Teaching Hospital, Parklane, Enugu, Nigeria,; 3 Department of Pathology, Enugu State University of Science and Technology, College of Medicine/Teaching Hospital, Parklane, Enugu, Nigeria,; 4 Departments of Health Administration and Management, University of Nigeria Enugu Campus, Enugu, Nigeria,; 5 Department of Community Medicine, University of Nigeria Teaching Hospital (UNTH), Enugu, Nigeria,; 6 Department of Obstetrics and Gynaecology, University of Nigeria Teaching Hospital (UNTH), Enugu, Nigeria

**Keywords:** Awareness, prevalence, cervical cancer screening, premalignant lesions

## Abstract

**Introduction:**

the prevalence of cervical squamous intraepithelial lesion is not well appreciated in most low-income countries. The study aimed to determine the level of awareness, prevalence and the pattern of squamous intraepithelial lesions and predictors for abnormal Pap smear reports (development of pre-malignant lesions of the cervix) among women attending various clinics in a tertiary health facility in Enugu, Nigeria.

**Methods:**

a cross-sectional study of 207 female patients attending various clinics of Enugu State University Teaching Hospital, Parklane, Enugu between June and August 2017 was undertaken. Structured interviewer-administered questionnaires were used for data collection while cervical smears were collected from the patients and sent for cytology. Data analysis was done using the Statistical Package for the Social Sciences (SPSS) version 22.0. The results were presented as means, standard deviations, frequencies and proportions. Pearson´s Chi-square test was used to test for associations between categorical variables and statistical significance was set at a p-value of < 0.05.

**Results:**

the levels of awareness of cervical cancer and the screening methods among the respondents were 76.8% and 36.7% respectively. The overall knowledge of cervical cancer and its screening was poor (6.8% and 29.0% respectively). The prevalence of pre-malignant lesions of the cervix among the respondents was 15.0% with low grade squamous intraepithelial lesion (LGSIL) having the highest frequency (38.7%). Among all the other risk factors for the development of premalignant lesions of the cervix among the respondents, a report of abnormal pap (positive) smear report was significantly associated with only age ≥35 years (χ^2^=5.723; p=0.017). The same age of 35 years and above also correctly predicted abnormal Pap smear reports among other factors (AOR = 3.02, 95% CI = 1.16 - 7.89, p = 0.024).

**Conclusion:**

the awareness of cervical cancer and cervical cancer screening was high but the overall knowledge on cervical cancer and its screening was very poor among the respondents. The prevalence of pre-malignant lesions of the cervix was high, and the commonest abnormal smear was LGSIL. Only age 35 years and above correctly predicted the occurrence of abnormal Pap smear reports among the respondents.

## Introduction

Cancer of the uterine cervix is the fourth most common cancer among women worldwide and the second leading cause of female cancer in Nigeria [1]. An estimate of 570,000 new cases was recorded worldwide in 2018 and out of 311,000 deaths recorded yearly, 85% of them occurred in low and middle income countries [1]. In Nigeria, an estimated 14,943 new cervical cancer cases were diagnosed annually and 10,403 persons died from the disease [2]. The crude mortality rate worldwide was 7.6 per 100,000 women per year, while that of Nigeria was 10.8 per 100,000 women per year [2]. The incidence of cancer of the cervix may have grown because of the increased prevalence of human immunodeficiency virus infection, as it has been reported that the disease is more common among patients with human immunodeficiency virus/ acquired immune deficiency syndrome (HIV/AIDS) [3, 4].

Cervical cancer is the only cancer that is almost completely preventable by safe, simple and inexpensive methods [5]. Sadly, most women especially those in developing countries like Nigeria present for treatment in very advanced stages of the disease, posing a great challenge. Treatment facilities at this advanced stage are most times unavailable. Therefore, early detection can easily reduce the number of advanced cervical cancer cases, the financial burden of treating advanced cases and the loss of life secondary to the disease. Various screening methods for early detection of cervical cancer include the Papanicolaou test (cervical Pap smear), liquid-based cytology, visual inspection with acetic acid (VIA), visual inspection with Lugol's iodine (VILI), and human papillomavirus testing. The death rates from cervical cancer have reduced significantly in the developed world due to the availability of these screening tests especially Pap smears. A cervical Pap smear is the most successful in reducing the incidence and mortality from cervical cancer in developed countries [5]. The Pap smear test is the most successful cancer screening technique in history [6]. It was named after its inventor, Dr. George Nicholas Papanicolaou whose work became widely known and accepted in 1943 when he published the book, “Diagnosis of uterine cancer by the vaginal smear” [6].

Unfortunately, despite the greatest burden of cervical cancer in developing countries especially Nigeria, and the numerous benefits of Pap smear, there is no available national cervical cancer screening programme and no effort is being made towards having an effective cancer screening policy at all levels of healthcare in Nigeria. Barriers to screening in developing countries have been identified, including competing healthcare needs, lack of awareness of the availability of screening methods, limited human and financial resources, poorly developed healthcare services, illiteracy, lack of women empowerment with its attendant poor health-seeking behaviour, war and civil strife, as well as widespread poverty [7].

Despite several efforts towards removing these barriers and improving the participation of women in cervical cancer screening little or no significant results in many places have been recorded. The uptake of Pap smear tests remains low in these developing countries. A retrospective study on the participation in highly subsidized cervical cancer screening by women in Enugu, South-East Nigeria showed that less than 1% of the targeted women population participated [8]. In a cross-sectional study by Nwankwo *et al*. on cervical cancer screening among certified nurses in Enugu: knowledge, attitude and uptake of Pap smear test, it was discovered that although the nurses had good knowledge of cervical cancer screening, their uptake of Pap smear test was very low as only 12.2% of the respondents had ever done Pap smear test [9]. A similar finding was reported in a similar recent study involving nurses in Enugu, Nigeria [10]. Compulsory screening of women at points of contact with them in the hospital has been difficult to implement in most hospitals.

There is a need to devise appropriate educational programmes that will inform health workers and women on the increased need for correct implementation of the Pap smear test and participation of women in the test. The various clinics in the hospital provide good entry points for women to be screened for pre-malignant and malignant lesions of the cervix. Enugu State University Teaching Hospital (ESUTH) records a large number of patients being referred to the hospital. This, therefore, provides a good opportunity for cervical cancer screening among these women using Pap smear. This study was aimed at determining the level of awareness, prevalence and the pattern and predictors of pre-malignant lesions of the cervix among women attending various clinics in ESUT Teaching Hospital Parklane, Enugu, and also, to determine the predictors for the occurrence of positive (abnormal) Pap smear reports among the respondents. The findings generated from this study would serve as a baseline value for the hospital on the prevalence of cervical premalignant lesions among women attending the hospital. These findings can also help in attracting the needed funding towards cervical cancer screening in the hospital and will equally contribute to the existing knowledge of cervical premalignant lesions in Enugu, Nigeria. Also, the findings would help the hospital management and other similar hospitals in Nigeria, policymakers and other stakeholders plan on how to remarkably improve the uptake of Pap smear tests in our environment and reduce the high incidence of cervical cancer in our environment.

## Methods

**Study design:** this was a cross-sectional analytical study.

**Study setting:** the study was conducted in Enugu at the Enugu State University Teaching Hospital (ESUTH) Parklane, Enugu, among female patients attending the various clinics in the hospital. Data were collected within a period of three months, from June to August 2017. Enugu State University Teaching Hospital is a tertiary institution located in the heart of the city of Enugu and is a major referral centre for health issues requiring specialist care. Patients that are referred from other centres are seen at the various specialist clinics including the Gynaecology Clinic, the Medical Out-Patient (MOP) Clinic, the Surgical Out-Patient (SOP) Clinic, and the Eye Clinic. New patients who come to the hospital without any referral are first seen at the General Out-Patient Department (GOPD) where they are attended to and when necessary, they may be referred to the specialist clinics. Pregnant women are seen at the antenatal clinic. Very unstable patients/emergencies are treated at the accident and emergency unit. All the clinics run every day from Monday to Friday mostly between 8.00 a.m. and 4.00 p.m.

**Study population:** the study population was all the female patients attending the General Outpatient Department and other specialist clinics in Enugu State University Teaching Hospital (ESUTH) Parklane, Enugu.

**Inclusion/exclusion criteria:** all sexually active women who attended the clinics were included. The study excluded those who were menstruating, had ongoing vaginal bleeding or obvious cancer of the cervix, or those who had a total abdominal hysterectomy in the past. Also, patients who practiced douching and had done so within the previous 48 hours or had sexual intercourse within the same period were excluded.

**Sampling technique:** a multi-staged sampling technique was utilized in the selection of the study participants. At the first level, a convenient sampling technique was used to select all the clinics in the hospital. At the second level, a proportionate sampling technique was used to allocate the women to the various clinics based on the number of women that attended each clinic in the previous month. Lastly, using a systematic sampling method, a consenting participant was selected after every other participant. Questionnaires were administered to consenting qualified women until the sample size was reached. Those who refused to be recruited were excluded from the study.

**Data collection:** having met the inclusion criteria, the procedure was explained to each patient, and her consent was obtained. A structured interviewer-administered questionnaire was used to obtain information from the consenting participants, including their socio-demographic characteristics such as age, marital status, occupation, educational level, ethnicity and religion; their level of awareness of cervical cancer and Pap smear, and possible risk factors for premalignant and malignant lesions of the cervix. In the presence of a female chaperon, the participant was then taken to the cytology room for specimen collection. With the patient in a lithotomy position to expose the perineum, a disposable speculum was passed into the vagina to expose the cervix. Any obvious mucus discharge was gently cleaned with a dry swab. The Ayre's spatula was used to collect cells from the ectocervix which were smeared on the glass slide already labelled with a code number. The cytobrush was then inserted into the cervix to collect cells from the endocervix which were smeared on another labelled glass slide. Each of the glass slides was quickly dropped into a container containing 95% ethyl alcohol after smearing to fix the smear and avoid drying of the cells. Each container was labelled for proper identification. The specimen was sent to the pathologist for reporting. Two cytopathologists reported the slides and if there was any discrepancy in any slide, that particular slide was taken to a third cytopathologist for further reporting to serve as a tie-breaker.

**Outcome measures:** the primary outcome measure was the prevalence of abnormal Pap smear results among the participants. The secondary outcome measures were overall knowledge of cervical cancer and its screening among the participants.

**Sample size estimation:** a sample size of 212 participants was calculated for the study using the formula [11]:


$$N=\frac{z^{2}pq}{e^{2}}$$

Where Z = standard normal deviation at 95% confidence interval = 1.96, p = proportion or prevalence of abnormal Pap smear result from previous similar study in Enugu which was 15.2% [8], q = 1 - p = 0. 85, and e = precision limit = 0.05.

**Ethical approval and consent to participate:** ethical clearance certificate of approval number ESUTHP/C-MAC/RA/034/VOL.11/81 was obtained from the Research Ethics Committee of Enugu State University Teaching Hospital (ESUTH) Parklane, Enugu before proceeding with the study. An informed consent form was read and signed by each participant in the presence of the interviewer and a witness before each participant was recruited.

**Data analysis:** data collected was analyzed using the IBM Statistical Package for Social Sciences (SPSS) version 22 (SPSS® Inc., Chicago, IL, USA). Results were presented as means with standard deviations, rates and proportions, tables and figures. Cross tabulation using Chi-square was performed to establish relationships between variables. A P-value of <0.05 was taken as significant. Some participants with incomplete data were replaced. The STROBE guidelines for reporting observational studies were utilized in the writing of the manuscript [12].

## Results

**Baseline information:** a total of 212 women were recruited for the study from various clinics in Enugu State University of Science and Technology Teaching Hospital, Parklane, Enugu. Five samples out of this total were reported as inadequate. This gave a recovery rate of 97.6% as only 207 samples were analyzed. The respondents were drawn from six major clinics in ESUTH Parklane including Gynaecology Clinic, Family Planning Clinic, General Out-Patient Department, Medical Out-Patient Clinic, Surgical Out-Patient Clinic, and Eye Clinic. Women from the Antenatal Clinic were not included in the study because they refused to give consent due to fear of complications in their pregnancies. Details of the distribution of the respondents among the clinics were shown in Table 1.

**Table 1:** socio-demographic characteristics of respondents

Variable	Sub-category	Frequency (n=207)	Per cent
Age in years	21-30	43	20.8
	31-40	83	40.1
	41-50	45	21.7
	51-60	29	14.0
	>60	7	3.4
Educational level	Primary and below	16	7.7
	Secondary	52	25.1
	Tertiary	139	67.1
Occupation	Civil/public servants	81	39.1
	Petty trading	45	21.7
	Unemployed	35	16.9
	Skilled manual	13	6.3
	Others	33	15.9
Marital status	Single	27	13.0
	Married	168	81.2
	Others (widowed/divorced/separated)	12	5.8
Tribe	Igbo	201	97.1
	Others (Hausa/Yoruba/Urhobo)	6	2.9
Clinic	Gynaecology	83	40.3
	Family planning	52	25.2
	GOPD	50	24.3
	MOP	7	3.4
	SOP	4	1.9
	Eye clinic	10	4.9

GOPD: general outpatient department; MOP: medical out-patient; SOP: surgical out-patient

**Socio-demographic characteristics of the respondents:** the average age of the respondents was 39.35 ± 10.45 years while the age range was 21 - 71 years. Most of the respondents (162, 78.3%) were premenopausal and multiparous (82, 39.6%). Majority of the respondents studied were aged 31 - 40 years (83, 40.1%), had tertiary education (139, 67.1%); were civil/public servants (81, 39.1%), married (168, 81.2%), and of Igbo tribe (201, 97.1%). The details of the socio-demographic characteristics of the respondents were presented in Table 1.

**Knowledge of cervical cancer:** a higher proportion of respondents have heard of cervical cancer (159/207, 76.8%). The common sources of information were health workers (82/159, 51.57%), radio/TV (40/159, 25.16%), schools (18/159, 11.32%), newspapers (4/159, 2.51%), markets (1/159, 0.63%), internet/social media (15/159, 9.43%), books (10/159, 6.29%), friends/relatives (15/159, 9.44%) and from women meetings (3/159, 1.89%). Only 41/159 (25.78%) respondents who were aware of cervical cancer correctly identified that the human papillomavirus (HPV) causes cervical cancer while others believed it could be caused by bacteria (9/159, 5.66%) and fungi (3/159, 1.89%). On the mode of transmission, a majority (64/159, 40.25%), that was aware of cervical cancer knew that it was gotten through sexual contact. Other respondents believed other modes of transmission are possible such as through toilet/toiletries (11/159, 6.92%), handshakes (1/159, 0.63%), and through drinking water (1/159, 0.63%).

**Knowledge of treatment and prevention of cervical cancer:** about half of the respondents, (111/159, 69.8%) knew that cervical cancer can be treated, of these, 83/111 (74.8%) knew that chemotherapy, (18/111, 16.2%) and surgery (20/111, 18.0%) can be used for the treatment of cervical cancer. A majority (134/159, 84.7%) knew that cervical cancer can be prevented; with abstinence (15/134, 11.2%), keeping one sexual partner (45/134, 33.6%), vaccination (38/134, 28.4%), and early detection (10/134, 7.5%) as methods of prevention. Details were shown in Table 2.

**Table 2:** knowledge on treatment and prevention of cervical cancer

Variable	Sub-category		Frequency	Per cent
Heard of cervical cancer (n=207)	Yes		159	76.8
	No		48	23.2
Cervical cancer can be treated (n=159)	Yes		111	69.8
	No		48	30.2
Treatment modality/ways of treatment (n=111)	Drugs/chemotherapy	Yes	83	(74.8)
		No	28	(25.2)
	Prayers	Yes	18	(16.2)
		No	93	(83.8)
	Radiotherapy	Yes	18	(16.2)
		No	189	(83.8)
	Surgery	Yes	20	(18.0)
		No	91	(82.0)
	Herbal medications/ pouring of chemicals into the private part	Yes	3	(2.7)
		No	108	(97.3)
Cervical cancer can be prevented (n=159)		Yes	134	84.7
		No	25	15.3
Method of prevention (n=134)	Abstinence	Yes	15	11.2
		No	119	88.2
	Keeping one sexual partner	Yes	45	33.6
		No	89	66.4
	Vaccination	Yes	38	28.4
		No	96	71.6
	Stop smoking	Yes	4	1.9
		No	130	98.1
	Avoid early sexual intercourse	Yes	12	9.0
		No	122	91.0
	Prayers	Yes	2	1.5
		No	132	98.5
	Early detection	Yes	10	7.5
		No	124	92.5
	Public awareness	Yes	3	2.2
		No	131	97.8

**Knowledge of cervical cancer screening:** among all the respondents 36.7% (76/207) were aware of cervical cancer screening. The methods of cervical cancer screening the respondents were aware of, include: Pap smear (64.47%, 49/76), colposcopy (1.23%, 1/76), visual inspection with acetic acid (1.32%, 1/76) and both Pap smear and visual inspection with acetic acid (VIA) (2.63%, 2/76). On who should be screened, a majority (76.32%, 58/76) knew that sexually active women should be screened. Others believed that it was only elderly women (5.26%, 4/76), prostitutes (1.32%, 1/76), and other women (2.63%, 2/76) who should be screened.

**Knowledge and practice of Pap smear of the respondents:** a higher proportion of respondents have not heard about Pap smear (126/207, 60.9%). The commonest source of information was health workers (39/81, 48.1%) followed by radio/television (13/81, 16.0%). Only 70/81 (86.4%) knew that Pap smear is used for screening for cervical cancer while 25/207 (12.1%) had done Pap smear before. The major reasons for not doing Pap smear include: lack of information on Pap smear (117/126, 92.9%), lack of where to get screened (34/126, 27.0%) and not having money (13/126, 10.3%). The major reasons for doing Pap smear included; routine screening (8/81, 9.9%), vaginal discharge (6/81, 7.4%) and recommendation by a health worker (6/81, 7.4%). Of respondents that had done Pap smear, (24/25, 96.0%) were negative. Almost all (201/207, 97.1%) respondents (after little education) stated that Pap smear was necessary and would recommend it for others. Overall, it was only a small proportion of the respondents that have heard of cervical cancer, (14/159, 8.8%) had good knowledge of cervical cancer and management while 60/76, 78.9% of women who have heard of cervical cancer screening had good knowledge of cancer screening. Other details were shown in Table 3.

**Table 3:** knowledge and practice of Pap smear

Variables	Sub-category	Frequency (n=207)	Per cent
Heard about Pap smear	Yes	81	39.1
	No	126	60.9
Source of information (n=81)	Radio/TV	13	16.0
	Health workers	39	48.1
	School	10	12.3
	Newspaper	1	1.2
	Church	3	3.7
	Internet	2	2.5
	Books	2	2.5
	Friends	7	8.8
	Women meeting	1	1.2
	Health workers + any other	3	3.7
What Pap smear is used for	Detecting staph aureus	5	2.4
	Detecting cervical cancer	70	33.8
	Detecting bleeding from the vagina	1	0.5
	Detecting pelvic infection	6	2.9
	Don't know	124	60.4
Done Pap smear before	Yes	25	12.1
	No	182	87.9
Reason for not doing Pap smear	Don't know about screening	117	56.5
	Don't have money	13	6.3
	Don't know where to get screened	34	16.4
	Don't have a need for screening	5	2.4
	Have other things to take care of	7	3.4
	Cannot have cancer	1	0.5
	Afraid of getting a positive result	5	2.4
	Not applicable	25	12.1
Reason for Pap smear	Routine		8
	Vaginal bleeding	3	1.4
	Vaginal discharge	6	2.9
	Recommended by a health worker	6	2.9
	Screened in outreach programs	2	1.0
	Not applicable		182
Result of Pap smear	Negative	24	11.6
	Positive	1	0.5
	Not applicable	182	87.9
Recommend Pap smear to other women	Yes	201	97.1
	No	6	2.9

**Figure 1:**
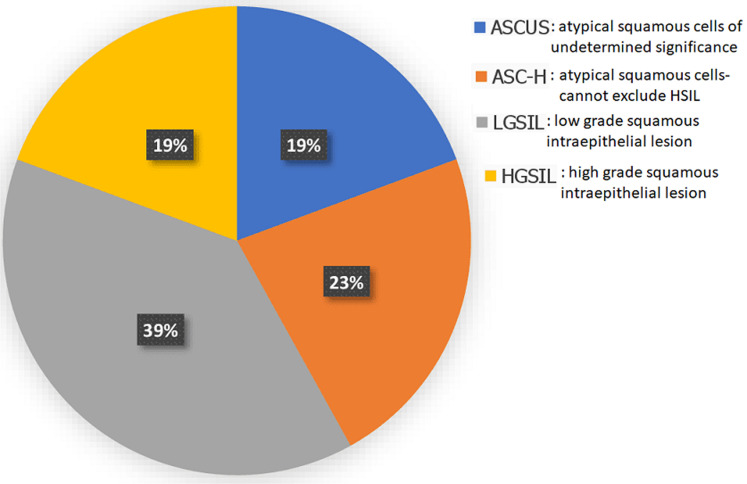
distribution of abnormal cervical smear results

**Prevalence and pattern of Pap smear findings of the respondents:** the prevalence of cervical SIL among the respondents was 15%. The prevalence of LGSIL was 38.7% while it was 19.35 for high grade squamous intraepithelial lesion (HGSIL) among women with abnormal Pap smear results. Other details are shown in Figure 1.

**Relationship between the identified risk factors for premalignant and malignant lesions of the cervix and the development of premalignant lesions:** the age of ≥ 35 years was significantly associated with the report of abnormal Pap smear results compared to age groups less than 35 years (χ^2^=5.723; p=0.017). There were no statistically (significant) associations of age at first sexual contact (χ^2^=0.756; p=0.384), number of partners (χ^2^=3.483; p=0.062), use of oral contraceptive pills (OCP) (χ^2^=0.136; p=0.712), substance use (cigarette/tobacco) (FT=1.00; p=0.850), number of deliveries (FT=2.539; p=0.147), and previous history of sexually transmitted infection (χ^2^=0.220; p=0.639) with the development of premalignant lesions among the respondents. The development of premalignant and malignant lesions of the cervix as shown by abnormal Pap smear results was only predicted by age groups as abnormal Pap smear reports were 3 times higher in age groups 35 years and above compared to women that were less than 35 years of age (AOR=3.02, 95% CI=1.16 - 7.89, p= 0.024). The details were shown in Table 4 and Table 5.

**Table 4:** relationship between the identified risk factors for premalignant and malignant lesions of the cervix and the development of premalignant lesions

Variable	Sub-category	Result of Pap smear n=207					
		Negative		Positive (abnormal)		χ^2^ test	p-value
		Frequency	Per cent	Frequency	Per cent		
Age groups (years)	<35	74	92.5	6	7.5	5.723	0.017
	≥35	102	80.3	25	19.7		
Age of first sex contact (years)	<20	65	82.3	14	17.7	0.756	0.384
	≥20	111	86.7	17	13.3		
Number of partners	1	60	78.9	16	21.1	3.483	0.062
	>1	116	88.5	15	11.5		
Use of OCP	Yes	24	82.8	5	17.2	0.136	0.712
	No	152	85.4	26	14.6		
Use of tobacco/cigarette	Yes	1	100.0	0	0.0	*1.00	0.850
	No	175	85.0	31	15.0		
Parity	Nulliparous	39	92.9	3	7.1	*2.539	0.147
	Parous	137	83.0	28	17.0		
STI	No STI	66	83.5	13	16.5	0.220	0.639
	STI	110	85.9	18	14.1		

* : Fisher's test

**Table 5:** predictors of the development of premalignant and malignant lesions of the cervix among the participants

Variable	Sub-group	Result of Pap smear (n=207)				P- value	**aOR (CI95%)	P-value
		Positive		Negative				
		Freq	%	Freq	%			
Age groups (years)	<35	74	92.5	6	7.5	0.017	3.02(1.16-7.89)	0.024
	≥35	102	80.3	25	19.7			
Parity	Nulliparous	39	92.9	3	7.1	*0.147	2.11(0.59-7.52)	0.248
	Parous	137	83.0	28	17.0			
Number of partners	One	60	78.9	16	21.1	0.062	0.459(0.21-1.01)	0.054
	≥Two	116	88.5	15	11.5			

## Discussion

This study was conducted to assess the awareness (knowledge) and prevalence of premalignant lesions of the cervix and predictors of abnormal Pap smear report among female patients seen in various clinics in our hospital. The study showed that the levels of awareness of cervical cancer and the screening methods among the respondents were 76.8% and 36.7% respectively. The overall knowledge of cervical cancer and its screening was poor (6.8% and 29.0% respectively). The prevalence of pre-malignant lesions of the cervix among the respondents was 15.0% with low grade squamous intraepithelial lesion (LGSIL) having the highest frequency (38.7%). Among all the other risk factors for the development of premalignant lesions of the cervix among the respondents, a report of abnormal pap (positive) smear report was correctly predicted by age groups as the occurrence of abnormal Pap smear report was 3 times higher in age ≥ 35 years compared to those < 35 years of age (AOR = 3.02, 95% CI = 1.16 - 7.89, p = 0.024).

The study finding of 76.8% on cervical cancer awareness among the respondents was higher than the 66.9% reported in another study in Zaria and 50.9% reported in Jos, both in Northern Nigeria [13, 14]. However, despite the relatively high level of awareness of cervical cancer, the overall knowledge of cervical cancer and its management was poor (6.8%). Again, the level of awareness of cervical cancer screening was quite low (36.7%) and only 12.1% of the respondents had done Pap smear in the past. The overall knowledge on cervical cancer screening was poor as only 29.0% of the respondents had good knowledge of cervical cancer screening. This finding is comparable to the result obtained in Onitsha, South-East Nigeria where the awareness of cervical cancer screening was 35.6% [15]. The low level of uptake of Pap smear in the history of the respondents observed in this study is similar to findings from studies in other parts of the country such as Jos (10.2%) [14] and Owerri (7.1%) [16]. Relatively higher uptake of Pap smear (34.6%) found in Ibadan was among female nurses (health workers) [17], although this is still very low compared to what is obtainable in developed countries where organized screening programmes are put in place, contributing to their low mortality rate from cervical cancer [18].

The reasons for not being screened provided by the respondents included lack of awareness of any screening modality available for cervical cancer, financial constraints, lack of awareness of where to get screened, belief in not needing screening or not being at risk, having other contending issues to take care of, and the fear of getting a positive result. These were similar to findings from previous studies in other places [14, 15, 19-21]. These findings suggest that accurate information about cervical cancer, its causes/risk factors, prevention and modes of treatment as well as the availability of screening tests for early detection needs to be made available to women through every means such as health talks in hospitals, mass media, churches/mosques, market places, and during women´s meetings. The government should set up effective screening services and make it available and affordable to women of reproductive age located in every part of the country including the rural areas. Also, non-governmental organizations and philanthropists could help in subsidizing the cost of obtaining cervical cancer screening so that every woman could have access to it. This will go a long way in reducing the high mortality rate associated with cervical cancer in our country.

The prevalence of premalignant lesions of the cervix in this study was 15%. This is similar to what was obtained in a previous study in Enugu by Obi *et al*. which was a 10-year retrospective study [8]. This result was quite higher than the 7.7% obtained in a hospital-based study in North-central Nigeria [22], and the 11.2% prevalence obtained from a previous study in Abakiliki [23]. The result buttresses the fact that the prevalence of premalignant lesions of the cervix is still high in this part of the world. This is further supported by the fact that abnormal Pap smear results were obtained from the respondents that came from the various clinics in the hospital who had no symptoms suggestive of any disease of the cervix. There is therefore an urgent need to implement the previous suggestion by Dim *et al*. that routine cervical cancer counselling and screening with the opt-out option should be offered to every eligible woman attending the outpatient clinics [24]. This will go a long way to reduce the incidence and burden of cervical cancer in our environment due to early detection and treatment. The result of our study showed that low grade squamous intraepithelial lesion (LGSIL) was more prevalent (38.7%) among the types of abnormal smear seen. This is comparable to though lower than the findings in Maiduguri by Bukar *et al*. where CIN 1 constituted 53.7% of the premalignant lesions seen [25].

The risk factors for premalignant and malignant lesions of the cervix considered in this study included age at first sexual contact, parity, multiple sexual partners, sexually transmitted infection, use of oral contraceptive pills (OCP), and use of cigarette or tobacco. It was only age ≥ 35 years that was significantly associated and predicted the occurrence of abnormal Pap smear reports among the respondents. The findings from the study also showed a higher prevalence of premalignant lesions of the cervix among those who had their first sexual contact before the age of 20 years, those that used OCP and grand multiparous women although the findings were not statistically significant (p>0.05). Similarly, there was no significant association between a previous history of sexually transmitted infection, the number of sexual partners, use of cigarettes or tobacco and development of premalignant lesion of the cervix from the study (p>0.05). This is at variance with the findings by Dare *et al*. in which 82.5% of those who developed premalignant lesions of the cervix had a previous history of sexually transmitted infection while the subjects using oral contraceptive pills had a higher rate of premalignant lesions (n = 51, 44.7%) than those using an intrauterine device (n = 21, 18.4%) or injectable (n = 11, 9.7%) [26]. The difference in the findings could be from the number of subjects studied since other studies have established that a previous history of sexually transmitted infection, prolonged use of oral contraceptive pills and cigarette smoking are risk factors for the development of premalignant lesions of the cervix [27, 28]. A prospective multi-centre study with a large sample size is therefore needed to help deeply explain the previously documented association among this study population.

**Limitations:** there could be recall bias as patients may find it difficult to remember past events in their lives. This was reduced by making the information collection method interviewer assisted. The materials for the Pap smear were also difficult and expensive to source.

## Conclusion

The awareness of cervical cancer/cervical cancer screening was high although the overall knowledge on cervical cancer management and its screening was very poor among the respondents. The prevalence of pre-malignant lesions of the cervix was high, and the commonest abnormal smear was LGSIL. The only factor that was associated with and predicted the occurrence of abnormal Pap smears reports among the respondents was age as abnormal Pap smear was 3 times higher in age ≥ 35 years compared to those that are younger.

**Recommendation:** the government and other stakeholders should make spirited efforts to equip women with all the necessary information about cervical cancer and establish effective screening programmes that will be available and affordable to reduce the burden of cervical cancer in our environment. Routine screening of all female patients attending all clinics in the hospital should be implemented in all secondary and tertiary hospitals in Nigeria to increase the opportunity of women to screen themselves for cervical cancer.

### What is known about this topic

- *Cancer of the uterine cervix is one of the leading causes of female cancer globally and early detection can reduce the number of advanced cervical cancer cases among women;*
- *The number of women presenting themselves in gynaecological clinics of our hospitals for cervical cancer screening has remained low in low-income countries;*
- *The prevalence of cervical squamous intraepithelial lesions is not appreciated in most low-income countries*.

### What this study adds

- *The overall knowledge of cervical cancer and its screening was poor, 6.8% and 29.0% respectively;*
- *The prevalence of pre-malignant lesions of the cervix among the respondents in the various clinics of the study site was 15.0% with low grade squamous intraepithelial lesion (LGSIL) having the highest frequency (38.7%);*
- *Age ≥ 35 years was significantly associated and correctly predicted abnormal Pap smear results among the respondents*.
